# Draft genome of *Aeromonas veronii* DFR01, the inactivated bacterial agent in a fish oral vaccine against motile aeromonad septicemia

**DOI:** 10.1128/mra.00733-24

**Published:** 2024-10-14

**Authors:** Anacleto M. Argayosa, Paul Christian T. Gloria, Vina B. Argayosa, Mary Nia M. Santos

**Affiliations:** 1 Trinity University of Asia, Quezon City, Philippines; 2 Natural Sciences Research Institute, University of the Philippines, Quezon City, Philippines; 3 Aquaculture Research and Development Division, National Fisheries Research and Development Institute, Quezon City, Philippines; Montana State University, Bozeman, Montana, USA

**Keywords:** *Aeromonas veronii*, draft genome, genomics, motile aeromonad septicemia, oral vaccine, Nile tilapia

## Abstract

*Aeromonas veronii* DFR01 was isolated from Nile tilapia (*Oreochromis niloticus*) infected with motile aeromonad septicemia and has been developed as an oral vaccine. We report a 4.538-Mb draft genome with 99.4% BUSCO and 98.85% CheckM completeness.

## ANNOUNCEMENT

*Aeromonas* sp. is a gram-negative rod bacterium that causes severe motile aeromonad septicemia (MAS), a significant threat to the tilapia farming industry worldwide (1). The isolation of *Aeromonas* sp. DFR01 emphasizes the importance of monitoring and controlling the bacterial diseases that affect aquaculture. To combat the disease, a fish oral vaccine against MAS was developed in the Philippines (2, 3). The fish oral vaccine was prepared using inactivated *Aeromonas* sp. DFR01, which has previously decimated the population of test fingerlings (juvenile fish, 6 ± 0.5 g) in the tank trials, showing ruptured abdomen, fin rot, skin ulceration, discoloration, and bulging eyes (4). The vaccine was tested in tank trials at SEAFDEC/AQD in Tigbauan, Iloilo, Philippines, and the results showed 53%–67% relative percent survival rates 2 weeks post-infection, indicating the vaccine’s considerable effectiveness in protecting tilapia against MAS (4). Thus, it is imperative to do a genomic analysis of the *Aeromonas* sp. DFR01 to confirm its identity.

The isolate *Aeromonas* sp. DFR01 was collected from diseased Nile tilapia (*Oreochromis niloticus*) in September 2011 from Binangonan, Rizal, Philippines (4). The vaccine strain was maintained in nutrient agar, L-dried for preservation, and deposited at the UP NSRI Culture Collection with isolate code UPCC 1376. For DNA extraction, the strain was subcultured in Luria Bertani (LB) broth at 30°C at 220 rpm for 16–18 hours. LB cultures were subjected to genomic DNA isolation using G-Spin Total DNA Extraction Kit (iNtRON Biotechnology, Korea) following manufacturer’s instructions. The extracted DNA was sent to SeqCenter, LLC (Pittsburgh, PA, USA) for sequencing using an Illumina NovaSeq X Plus sequencer with 151 bp pair-ended reads, from Illumina DNA Prep kit-prepared libraries. All bioinformatics tools were used under default parameters unless specified otherwise. Raw reads were quality trimmed to *Q*20 in Trimmomatic v0.36 (5). Unicycler v.0.5 (6) was used to generate an assembly. Assembly quality was evaluated using QUAST v.5.2.0 (7). Genome completeness and contamination were evaluated using BUSCO v.5.5 (8) and CheckM v.1.2.3 (9). Taxonomic identity was inferred using Insert Genome Into SpeciesTree v2.2.0 in KBase (10), which utilizes FastTree 2.0 (11) to place genomes into a phylogenomic tree containing 49 clusters of orthologous genes as markers. Finally, annotations were generated using RAST v.1.073 (12) and Prokka v1.14.5 (13).

**Fig 1:**
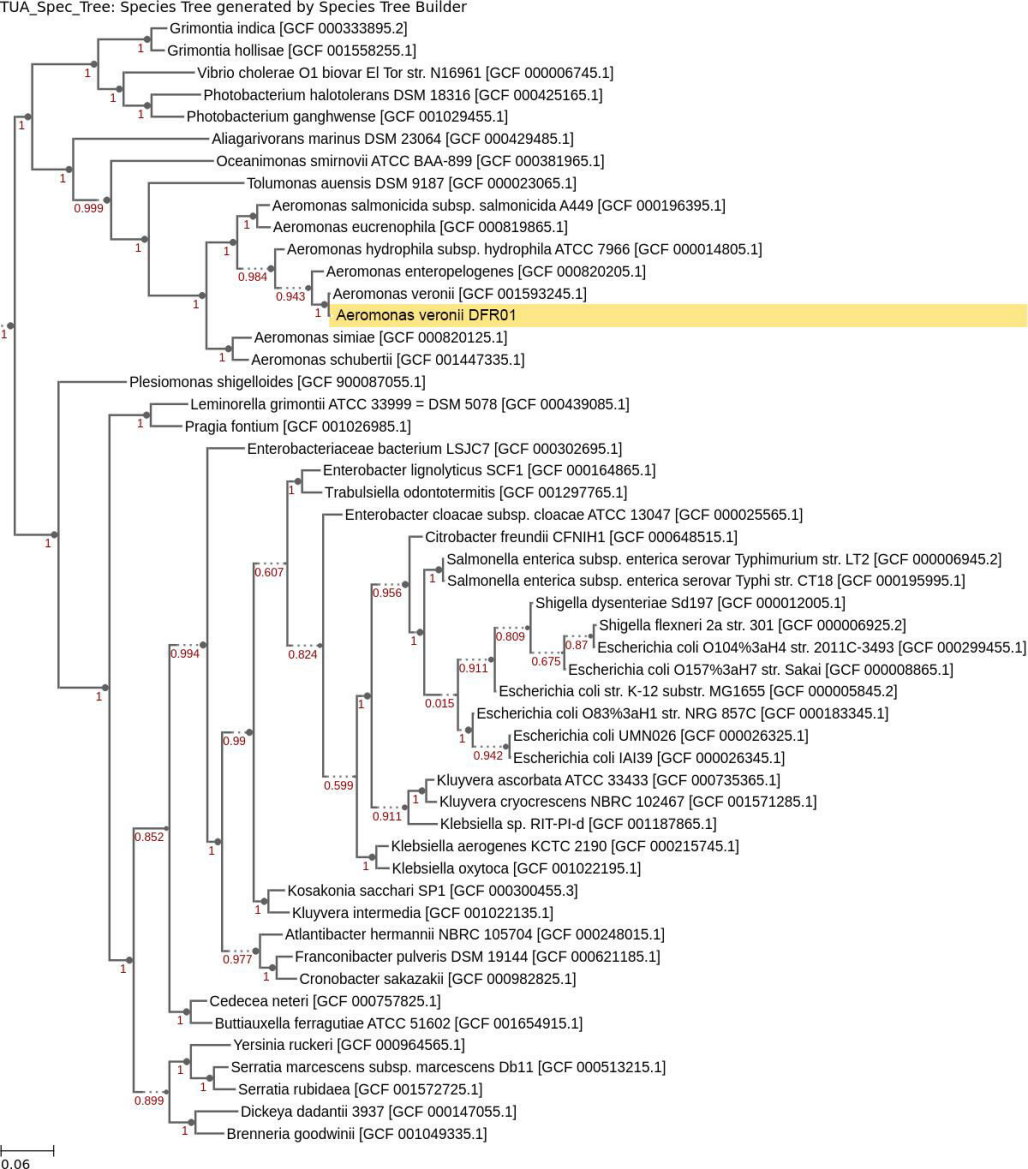
Phylogenetic tree containing *Aeromonas veronii* DFR01 along with 50 other RefSeq genomes, which were automatically picked as closest neighbors using Insert SpeciesTree v.2.2.0 in KBase. The tree incorporates phylogenetic information from 49 core, universal genes defined by Clusters of Orthologous Groups gene families. The complete list of 49 markers along with alignment and tree-building algorithms can be found here. Node values represent bootstrap support.

Sequencing produced 8,968,628 raw reads. Genome assembly yielded a size of 4,538,290 bp, GC content of 58.76%, with *N*_50_ of 167,321, *L*_50_ of 7,100× genome coverage, and 74 contigs in total. The largest contig is about 485 kbp in length. BUSCO analysis indicated 99.4% completeness, while CheckM indicated 98.85% completeness and 1.1% contamination. Insert SpeciesTree placed *Aeromonas* sp. DFR01 within a single clade to *Aeromonas veronii* [GCF 000820125.1] (Fig. 1). Thus, we designate the isolate as *Aeromonas veronii* DFR01. Prokka and RAST predicted 4,153 and 7,864 genes, respectively.

## Data Availability

This genome project has been registered at NCBI under accession number PRJNA1122951. Raw reads were deposited at the NCBI Sequence Read Archive (SRA) under the accession number SRR29367444. The genome sequence has been deposited at GenBank under the accession number JBEHWV000000000.1 with the assembly accessioned under ASM4023974v1. Lastly, annotations using RAST and Prokka have been uploaded in Xenodo.
